# Does wing reduction influence the relationship between altitude and insect body size? A case study using New Zealand's diverse stonefly fauna

**DOI:** 10.1002/ece3.3713

**Published:** 2017-12-12

**Authors:** Graham A. McCulloch, Jonathan M. Waters

**Affiliations:** ^1^ School of Biological Sciences The University of Queensland Brisbane Qld Australia; ^2^ Department of Zoology University of Otago Dunedin New Zealand

**Keywords:** alpine, Bergmann's rule, Plecoptera, wing loss

## Abstract

Researchers have long been intrigued by evolutionary processes that explain biological diversity. Numerous studies have reported strong associations between animal body size and altitude, but insect analyses have often yielded equivocal results. Here, we analyze a collection database of New Zealand's diverse endemic stonefly fauna (106 species across 21 genera) to test for relationships between altitude and plecopteran body size. This insect assemblage includes a variety of wing‐reduced (26 spp) and fully winged (80 spp) taxa and covers a broad range of altitudes (0–2,000 m). We detected significant relationships between altitude and body size for wing‐reduced, but not fully winged, stonefly taxa. These results suggest that, while the maintenance of flight apparatus might place a constraint on body size in some fully winged species, the loss of flight may free insects from this evolutionary constraint. We suggest that rapid switches in insect dispersal ability may facilitate rapid evolutionary shifts across a number of biological attributes and may explain the inconsistent results from previous macroecological analyses of insect assemblages.

## 1 INTRODUCTION

Biologists have long been intrigued by associations between abiotic and biotic variation. Notably, numerous studies have found that both intraspecific and interspecific biological variation can be linked to factors such as altitude and latitude (see Gaston, Chown, & Evans, 2008; Mayr, 1956). Bergmann (1847), for instance, noted that animal body size tends to increase gradually with increasing distance from the equator and also with increased altitude. Such “Bergmann clines” have been interpreted as responses to temperature, with the increased surface‐to‐volume ratios of larger individuals helping to conserve heat in cold climates (Atkinson & Sibly, 1997; Bergmann, 1847). Subsequently, analyses of body‐size variation have remained a key topic in animal biology, as this fundamental characteristic can strongly influence a range of biotic traits, including life span, clutch size, growth rate, and even biogeographic distributions (Blackburn & Gaston, 1994, 1999, 2001).

While Bergmann's rule originally described trends within endotherm species (Bergmann, 1847; Blackburn, Gaston, & Loder, 1999; James, 1970; Mayr, 1956), his hypothesis has subsequently, and controversially, been extended to both interspecific patterns and to ectotherm taxa (see Meiri, 2011; Salewski & Watt, 2017; Watt, Mitchell, & Salewski, 2010). Recent systematic reviews indicate that numerous bird and mammal taxa display both intraspecific and interspecific Bergmann clines (Clauss, Dittmann, Müller, Meloro, & Codron, 2013; Meiri & Dayan, 2003), whereas such clines are apparently relatively rare in insects (see Blanckenhorn & Demont, 2004; Chown & Gaston, 2010; Shelomi, 2012). Indeed, in insects, “converse” Bergmann clines (i.e., body size decreasing with decreasing temperature) have been identified just as commonly as conventional Bergmann clines (Shelomi, 2012). Such converse clines could perhaps be driven by the relatively short growth seasons and limited food resources available in cooler environments (see Mousseau (1997)).

In broad terms, conformity (or otherwise) to Bergmann's rule has been suggested to reflect the idiosyncrasies of both insect biology and study design (Shelomi, 2012). For instance, Levy and Nufio (2015) showed that two short‐winged grasshopper species displayed converse Bergmann clines across an elevation gradient, whereas two long‐winged taxa displayed no such pattern. These intriguing contrasts suggest that dispersal ability might influence conformity to Bergmann's rule, although the generality of this pattern has yet to be tested. Ideally, this hypothesis should be assessed using a diverse but phylogenetically coherent assemblage of fully winged and wing‐reduced insect species.

New Zealand (NZ) is an isolated landmass that hosts numerous endemic insect radiations, including a diversity of both fully winged and wing‐reduced lineages, with wing‐reduced lineages particularly common in NZ's alpine habitats (Goldberg, Trewick, & Paterson, 2008; McLellan, 2006; Patrick, 2003; Trewick & Wallis, 2001). The presence of a number of independently wing‐reduced clades makes NZ a potentially informative system for assessing how alpine adaptation and loss of dispersal ability can influence insect biogeography (Dussex, Chuah, & Waters, 2016; McCulloch, Wallis, & Waters, 2009, 2010, 2017; Wallis, Waters, Upton, & Craw, 2016). Wing reduction and loss are particularly common in NZ's alpine stoneflies (Plecoptera), with at least 26 of the 106 described endemic species wing‐reduced (McLellan, 2006; Patrick, 2003). Moreover, the phylogenetic distribution of these 26 wing‐reduced taxa within the species‐rich Gripopterygidae clade indicates numerous independent losses of flight (McCulloch, Wallis, & Waters, 2016). While some alpine genera are completely wing‐reduced (e.g., *Holcoperla*,* Apteryoperla*,* Vesicaperla*), others include both fully winged and wing‐reduced species (e.g., *Zelandobius*,* Taraperla;* see McLellan (2006)). The extent of wing reduction can vary even within species (wing dimorphism), with some typically fully winged species (e.g., *Zelandobius foxi*,* Zelandoperla fenestrata*) occasionally having wing‐reduced upland populations (Dussex et al., 2016; McCulloch et al., 2009; McLellan, 1991, 1999).

Many of NZ's wing‐reduced plecopteran lineages have been recorded predominantly at high altitudes, whereas fully winged species can occur from sea level to 2,000 m. The mean recorded altitude for individual taxa ranges from 100 m (*Spaniocercoides watti*) to 1,696 m (*Holcoperla magna*; Table S1). Body size also varies greatly among New Zealand Plecoptera, with an almost 10‐fold difference in body length between the smallest (*Spaniocercoides jacksoni* = 4 mm; McLellan (1991)) and largest (*Holcoperla magna* = 38 mm; McLellan (1983)) species. The fauna's wide variation in both body size and altitude provides an opportunity to test for associations between these parameters. Additionally, the presence of numerous fully winged and wing‐reduced lineages offers potential to assess the effects of dispersal ability on conformity to Bergmann's rule. In the current study, we undertake biogeographic and morphological analyses of NZ's entire plecopteran fauna to (1) test whether wing reduction is associated with increased body size, (2) test for correlations between body size and altitude, and (3) assess whether the loss of dispersal ability (wing reduction) influences the relationship between body size and altitude.

## 2 METHODS

Collection records for all NZ stonefly species (106 species; 7,000 species/locality records) were obtained from the Stoneflies of New Zealand database (http://stoneflies.org.nz/), including species from the NZ's sub‐Antarctic Islands. Records from duplicate locations were removed, and the mean altitude for each species was calculated (Table S1). Body length data for all species were obtained from relevant literature (Appendix S1) and unpublished empirical data. All statistical analyses were performed in R 3.02 (R Core Team, 2013). Female and male body lengths were significantly correlated (*r* ^2^ = .930, *p *<* *.001; Figure S1), so female data were used as a surrogate for both.

**Figure 1:**
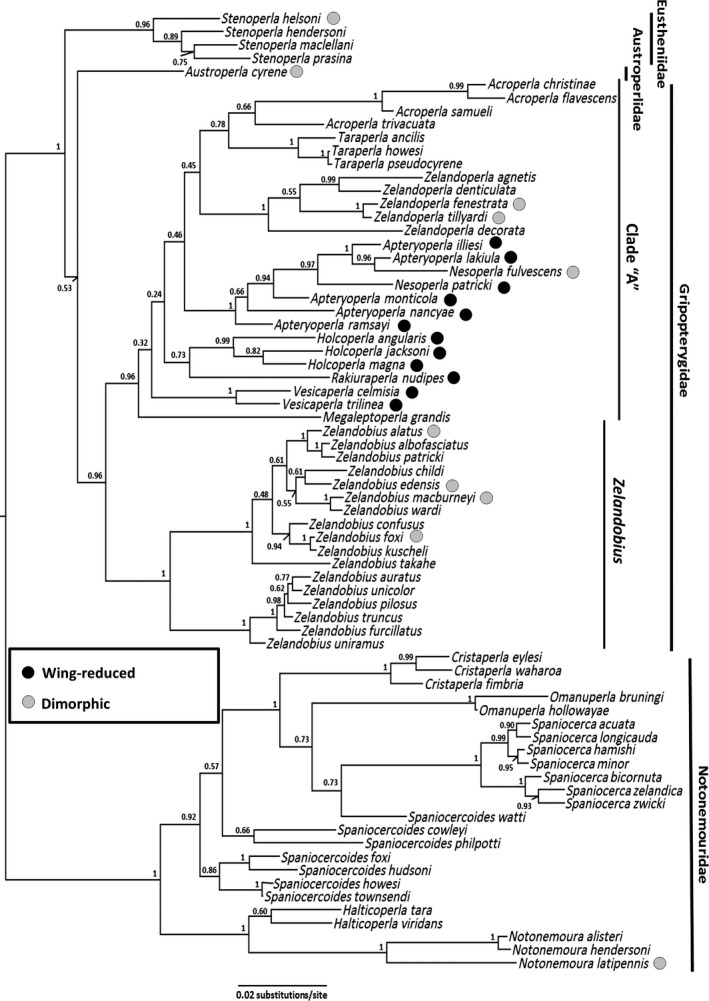
Bayesian maximum clade phylogeny of NZ Plecoptera based on three gene regions (18S, H3, COI; modified from (McCulloch et al., 2017)). The two divergent Gripopterygidae clades (Clade “A” and *Zelandobius*) are indicated, as is the distribution of wing‐reduced lineages

We compared the mean body size of the species in the families Eustheniidae, Gripopterygidae, and Notonemouridae and within two divergent Gripopterygidae clades (Clade “A” and *Zelandobius*; Figure 1). To account for phylogenetic nonindependence (e.g., given significant size differences among the three families (see below)), we subsequently focused on Clade “A” Gripopterygidae (which contains the 25/26 of NZ's wing‐reduced lineages) when assessing the relationship between body size and wing reduction. We compared the mean body lengths and mean altitudes of wing‐reduced and fully winged species and assessed the significance of any differences using a one‐tailed *t* test.

We estimated Pearson's correlation coefficients between mean body length and mean altitude for all stoneflies from the NZ mainland, as well as for the families Gripopterygidae and Notonemouridae, and for the genus *Zelandobius* and Clade “A” Gripopterygidae (Figure 1). To assess whether wing reduction affected the correlation, we again focused on Clade “A” of Gripopterygidae (Figure 1). Pearson's correlation coefficients were estimated for (1) all species in this clade, (2) only the wing‐reduced species in the clade, and (3) only the fully winged species in the clade.

## 3 RESULTS

**Figure 2:**
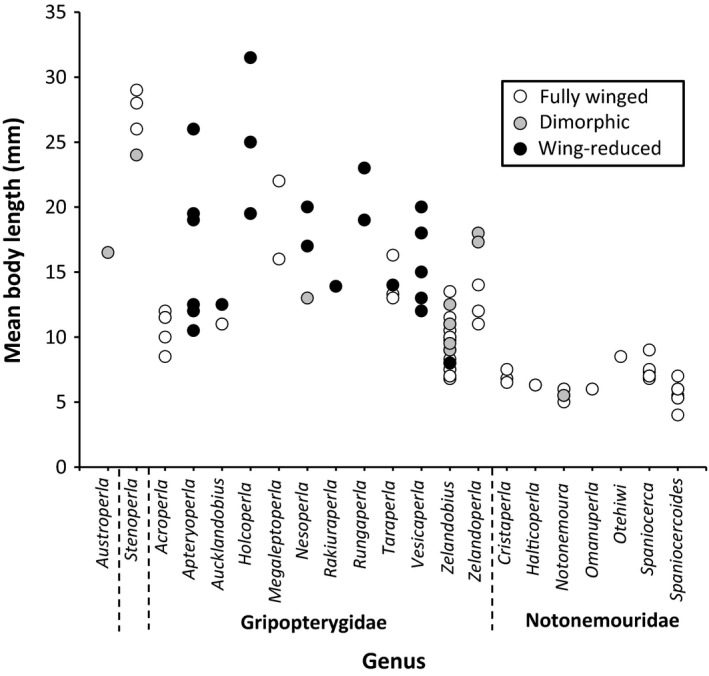
Mean body length and adult wing phenotype (fully winged, wing‐reduced, dimorphic) for all NZ stonefly species

Species within family Eustheniidae (Antarctoperlaria; mean length = 26.8 ± 2.2 mm) were significantly larger (*t*_76_ = 4.37, *p *<* *.001) than those from Gripopterygidae (Antarctoperlaria; mean length = 13.9 ± 5.8 mm) and family Notonemouridae (Arctoperlaria; mean length = 6.3 ± 1.0 mm, *t*_31_ = 31.84, *p *<* *.001). Species within Gripopterygidae were significantly larger (*t*_101_ = 6.83, *p *<* *.001) than those within family Notonemouridae, and the former also exhibited a notably greater range of body lengths (Figure 2). This high variation in body size was also seen within genera such as *Aucklandobius* and *Apteryoperla*, which both exhibited almost threefold differences in body length between smallest and largest species (Figure 2). Within Gripopterygidae, species from Clade “A” (containing a mixture of wing‐reduced and fully winged species) were significantly larger (*t*_67_ = 6.73, *p *<* *.001) than those from the primarily fully winged *Zelandobius* clade (Clade “A” mean length 15.8 ± 5.0 mm, *Zelandobius* mean length 9.3 ± 1.7 mm).

Wing‐reduced species ranged in length from 8 mm (*Zelandobius brevicauda*) to 31.5 mm (*Holcoperla magna*), whereas fully winged species ranged from 4 mm (*Spaniocercoides jacksoni*) to 29 mm (*Stenoperla maclellani*; Figure 2). Overall, wing‐reduced NZ stonefly species (mean length = 17.1 ± 5.6 mm) were significantly larger (*t*_101_ = 5.66, *p *=* *.001) than fully winged taxa (mean length = 10.1 ± 5.2 mm). As these results might be driven by the relatively high proportion of large wing‐reduced taxa in family Gripopterygidae, and the lack of wing‐reduced species in Notonemouridae (which contains significantly smaller species), we restricted subsequent analyses to the Gripopterygidae Clade “A.” Notably, the 25 wing‐reduced species (mean length = 17.5 ± 5.4 mm) in this clade were significantly larger (*t*_38_ = 2.64, *p *=* *.012) than the 17 fully winged species (mean length = 13.6 ± 3.4 mm), supporting the hypothesis that wing loss leads to an increase in body size.

Fully winged species ranged in mean altitude from 100 m to 1,610 m, while wing‐reduced species ranged in mean altitude from 270 m to 1,696 m. Wing‐reduced species (mean altitude = 1,111 ± 382 m) were found at a higher mean altitude (*t*_97_ = 3.67, *p *<* *.001) than fully winged species (mean altitude = 799 ± 333 m).

**Figure 3:**
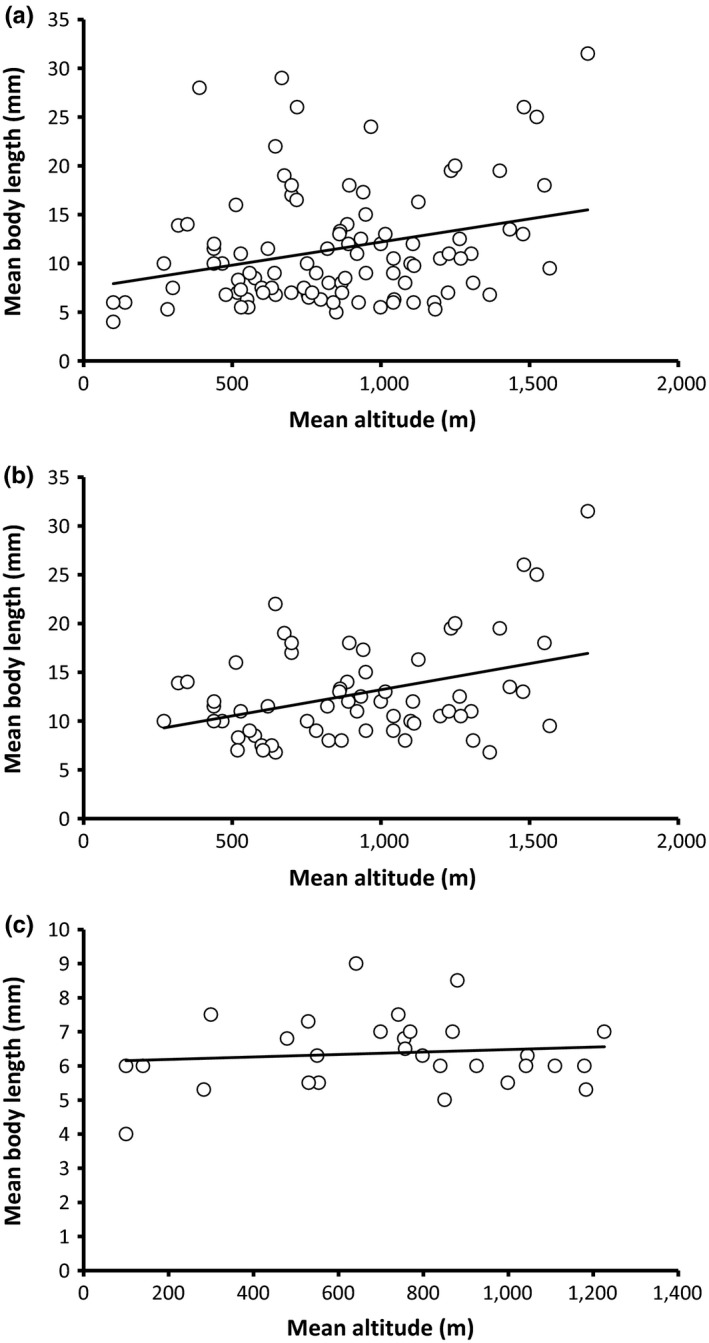
Mean altitude (m) versus mean body length (mm) for (a) all NZ Plecoptera (*r* ^2^ = .078, *p *=* *.005), (b) family Gripopterygidae (*r* ^2^ = .144, *p *=* *.002), (c) family Notonemouridae (*r* ^2^ = .013, *p *=* *.559)

**Figure 4:**
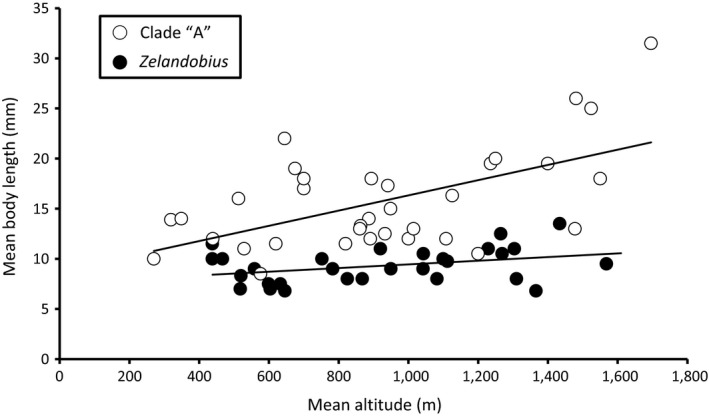
Mean altitude (m) versus mean body length (mm) for Clade “A” Gripopterygidae (*r* ^2^ = .328, *p *=* *.004; open circles) and *Zelandobius* (*r* ^2^ = .126, *p *=* *.060; black circles)

**Figure 5:**
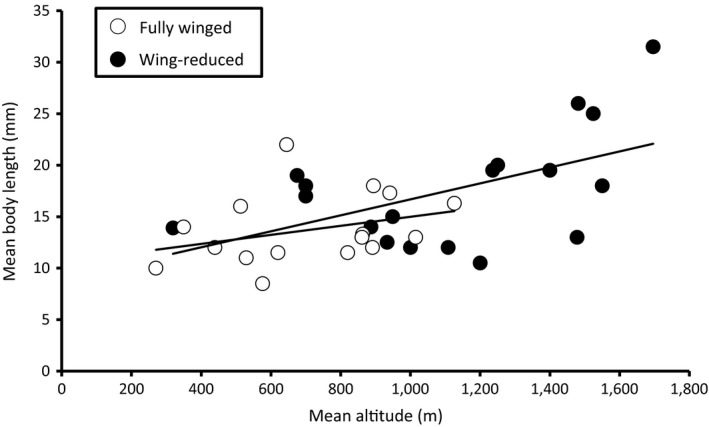
Mean altitude (m) versus mean body length (mm) for wing‐reduced Clade “A” Gripopterygidae (*r* ^2^ = .271, *p *=* *.027; black circles) and fully winged Clade “A” Gripopterygidae (*r* ^2^ = .103, *p *=* *.226; open circles)

Our analyses of the complete NZ plecopteran data set revealed a moderate (*r* ^2^ = .078) yet significant (*p *=* *.005) positive correlation between mean body length and mean altitude (Figure 3a), indicating that body size increases with altitude. A similar correlation (*r* ^2^ = .144, *p *=* *.002) was observed within Gripopterygidae species alone (which include a significant proportion of wing‐reduced species; Figure 3b), whereas no correlation was identified within the fully winged family Notonemouridae (*r* ^2^ = .013, *p *=* *.559; Figure 3c). No significant correlation (*r* ^2^ = .126; *p *=* *.060) was detected between mean body length and mean altitude within members of the primarily fully winged genus *Zelandobius* (Figure 4). In contrast, a strong (*r* ^2^ = .328) and significant (*p *=* *.004) correlation was also detected for Clade “A” Gripopterygidae taxa (containing a high proportion of wing‐reduced species). When the analyses were restricted to the wing‐reduced taxa in Clade “A,” a similar significant correlation was observed (*r* ^2^ = .271, *p *=* *.027; Figure 5). By contrast, no correlation was observed when only fully winged species were included in the analysis (*r* ^2^ = .103, *p *=* *.226; Figure 5), suggesting that the correlation between body size and altitude is being driven by the wing‐reduced taxa within this gripopterygid assemblage.

## 4 DISCUSSION

The current study examined biogeographic data for a diverse suite of endemic NZ stonefly taxa and found significant associations between body size and altitude across wing‐reduced taxa, but not for fully winged lineages. These findings suggest that loss of dispersal ability in alpine plecopteran lineages may be linked to a range of evolutionary shifts (see below). Wing‐reduced species were, however, found at higher mean altitudes than fully winged species, suggesting altitude, in additional to wing reduction, may play a role in conformity to Bergmann's rule.

Body‐size constraints associated with maintenance of flight ability may explain why fully winged plecopterans in this study show no relationship between altitude and size. By contrast, the loss of these constraints in wing‐reduced lineages might facilitate diversification with respect to body size. Notably, the majority of NZ's largest stonefly species are wing‐reduced (Figure 2). Additionally, wing‐reduced species from Clade “A” Gripopterygidae (Figure 1) are significantly larger than fully winged species. These results contrast with previous studies that suggested individuals from wing‐reduced stonefly populations are on average smaller than those from fully winged populations (Lillehammer, 1985; Loskutova & Zhiltzova, 2016; Zwick, 2000). These previous studies, however, focused primarily on species with a small degree of wing reduction (brachyptery), whereas many of the species in our study had significant wing reduction (vestigial wings, or complete wing loss). When reduced wings remain important for insect dispersal (e.g., plecopteran rowing and skimming behaviors), body size may remain under tight evolutionary constraints and inhibit the evolution of Bergmann clines.

Our differential findings for winged versus wing‐reduced stonefly lineages provide potential support for the recent hypothesis of Levy and Nufio (2015) that dispersal ability may influence conformity to Bergmann's rule. Specifically, variation in the direction and strength of Bergmann clines taxa may reflect differing constraints on the body sizes of flighted versus flightless taxa. Previous studies have noted significant correlations between insect body size and flight ability (Dudley, 2000; Helms, Godfrey, Ames, & Bridge, 2016; Lehmann, 2002), with the maintenance of dispersal ability (wings) suggested to present an important constraint on absolute body size (Frankino, Zwaan, Stern, & Brakefield, 2005). Once body size is no longer constrained by flight requirements, individuals may become larger, as increased body size has long been linked with increased fecundity (Darwin, 1883). We suggest that such switches may lead to the evolution of populations (and species) with larger body sizes.

The majority of previous intraspecific studies testing for links between insect size and altitude have failed to detect significant associations (Dillon, Frazier, & Dudley, 2006). Of the significant clines previously reported, there have been similar numbers of positive and inverse relationships (Chown & Gaston, 2010; Dillon et al., 2006; Shelomi, 2012). Results have been similarly mixed across broader taxonomic and ecological assemblages (Chown & Gaston, 2010; Dillon et al., 2006; Shelomi, 2012), although fewer such multispecies syntheses have been published. The direction of altitude‐size correlations seems to vary among different insect orders (Shelomi, 2012). Within stoneflies (Order Plecoptera), for instance, Bergmann clines (as identified this study) are more common than inverse Bergmann clines (Shelomi, 2012). Indeed, Bergmann clines have previously been reported within *Stenoperla prasina* (Winterbourn, Pohe, & Goldstien, 2017) and within several *Anacroneuria* species (Cressa, Maldonado, Segnini, & Chacon, 2008), with interspecific clines also reported within *Anacroneuria* (Tomanova & Tedesco, 2007). An inverse Bergmann cline was, however, reported within *Arcynopteryx dichroa* (Loskutova & Zhiltzova, 2016).

Higher temperatures at lower altitudes are often considered to provide a longer growing season which can lead to a larger final body size (Chown & Gaston, 2010; Mousseau, 1997), as total growing time and body size are correlated in insects (Roff, 1990). Species possessing the ability to extend their generation time across multiple years, however, could potentially overcome such constraints. Indeed, taxa from cooler climates often have extended generation times relative to taxa from warmer climates (Zeuss, Brunzel, & Brandl, 2017), and these differences in generation time (voltinism) have been demonstrated to significantly affect both the strength and direction of latitudinal size clines (Horne, Hirst, & Atkinson, 2015). Many of NZ's large alpine stonefly species are thought to be semivoltine (i.e., they may take multiple years to develop; McLellan personal communication), a factor that might help to explain their relatively large size.

It has been hypothesized that individuals from flightless populations are able to adapt to local optima without gene flow from continuous populations counteracting this process (Cassel‐Lundhagen, Kaňuch, Low, & Berggren, 2011). The identification of Bergmann's clines in fully winged species, but not wing‐reduced species, could potentially be a result of the greater isolation of populations within wing‐reduced species, particularly as wing‐reduced species are found at higher altitudes than their fully winged counterparts. This isolation is potentially amplified in many of NZ's wing‐reduced stonefly species as they also have exclusively terrestrial nymphs (McLellan, 1967), further limiting their dispersal potential (McCulloch et al., 2010). By contrast, substantial levels of gene flow among fully winged populations (McCulloch et al., 2009), coupled with the large geographic and altitudinal ranges (McCulloch et al., 2017), may preclude local diversification in fully winged taxa.

In summary, our findings suggest that evolutionary switches in dispersal ability may help to explain the biological diversification of insect assemblages (McCulloch et al., 2009, 2017). When plecopteran lineages are freed from the constraints imposed by maintenance of flight ability, they might have increased potential for adaptation to local conditions, perhaps leading to rapid shifts in body size. Future studies should further explore these processes in more detail at intraspecific scales.

## CONFLICT OF INTEREST

None declared.

## AUTHOR CONTRIBUTIONS

GM conceived the study, collated the data, and conducted the analyses. GM and JW wrote the manuscript.
