# Supplementary material for: Does wing reduction influence the relationship between altitude and insect body size? A case study using New Zealand's diverse stonefly fauna
Source: Ecol Evol. 2017 Dec 12;8(2):953–60. doi: 10.1002/ece3.3713 (PMC5773309; doi:10.1002/ece3.3713)
Supplement: Supplementary file 1 [file ECE3-8-953-s001.docx]

**Supporting Information**

**Table S1.** Body length, altitude, and adult wing phenotype database for all New Zealand Plecoptera.

| **Family** | **Species** | **Mean body length (range)** | | **Mean altitude** | **Wing phenotype** |
| --- | --- | --- | --- | --- | --- |
|  |  | **Females** | **Males** | **(range)** |  |
| Austroperlidae | *Austroperla cyrene* | 16.5 (13-20) | 12.5 (11-14) | 717 (5-1800) | Dimorphic |
| Eustheniidae | *Stenoperla helsoni* | 24 (24) | 19.5 (16-23) | 967 (470-1470) | Dimorphic |
|  | *Stenoperla hendersoni* | 26 (26) | 23 (20-26) | 718 (220-1350) | Fully-winged |
|  | *Stenoperla maclellani* | 29 (25-33) | 24 (22-26) | 667 (10-1520) | Fully-winged |
|  | *Stenoperla prasina* | 28 (21-33) | 21 (17-25) | 390 (3-1400) | Fully-winged |
| Gripopterygidae | *Acroperla christinae* | 12 (9.5-14.5) | 8.8 (8-9.5) | 891 (200-1300) | Fully-winged |
|  | *Acroperla flavescens* | 11.5 (11.5) | 10 (9-11) | 620 (300-1000) | Fully-winged |
|  | *Acroperla samueli* | 8.5 (8.5) | 10 (9-11) | 576 (100-880) | Fully-winged |
|  | *Acroperla spiniger* | 11.5 (11.5) | unknown | 820 (820-820) | Fully-winged |
|  | *Acroperla trivacuata* | 10 (9-11) | 8.8 (6.5-11) | 270 (15-1350) | Fully-winged |
|  | *Apteryoperla illiesi* | 19.5 (19-20) | 15.3 (15.3) | 1236 (730-1800) | Wing-reduced |
|  | *Apteryoperla lakiula* | 19 (19) | 17.5 (17.5) | 675 (650-700) | Wing-reduced |
|  | *Apteryoperla monticola* | 26 (23-29) | 21 (20-22) | 1481 (1432-1530) | Wing-reduced |
|  | *Apteryoperla nancyae* | 10.5 (10-11) | 12 (12) | 1200 (1200-1200) | Wing-reduced |
|  | *Apteryoperla ramsayi* | 12.5 (10-15) | 10.5 (9-12) | 933 (700-1100) | Wing-reduced |
|  | *Apteryoperla tillyardi* | 12 (12) | unknown | 1000 (1000-1000) | Wing-reduced |
|  | *Aucklandobius complementarius* | 11 (10-12) | 10 (9-11) | unknown | Fully-winged |
|  | *Aucklandobius gressitti* | 12.5 (12-13) | 11.5 (11-12) | unknown | Wing-reduced |
|  | *Aucklandobius kuscheli* | unknown | 24 (23-25) | unknown | Wing-reduced |
|  | *Aucklandobius turbotti* | 11 (10-12) | 13.5 (10-17) | unknown | Wing-reduced |
|  | *Holcoperla angularis* | 19.5 (19-20) | 18 (18) | 1400 (1000-1830) | Wing-reduced |
|  | *Holcoperla jacksoni* | 25 (21-29) | 25 (25) | 1524 (1160-1830) | Wing-reduced |
|  | *Holcoperla magna* | 31.5 (25-38) | 30.5 (29-32) | 1696 (1120-2000) | Wing-reduced |
|  | *Megaleptoperla diminuta* | 16 (16) | 13 (13) | 513 (5-1125) | Fully-winged |
|  | *Megaleptoperla grandis* | 22 (22) | 17.5 (17.5) | 645 (10-1640) | Fully-winged |
|  | *Nesoperla fulvescens* | 13 (13) | 11 (11) | 1015 (100-1600) | Dimorphic |
|  | *Nesoperla johnsi* | 20 (20) | 15 (15) | unknown | Wing-reduced |
|  | *Nesoperla patricki* | 17 (17) | 15.5 (14-17) | 700 (670-720) | Wing-reduced |
|  | *Rakiuraperla nudipes* | 13.9 (12.8-15) | 9 (9) | 319 (50-650) | Wing-reduced |
|  | *Rungaperla campbelli* | 19 (18-20) | 18 (18) | unknown | Wing-reduced |
|  | *Rungaperla longicauda* | 23 (22-24) | 20 (20) | unknown | Wing-reduced |
|  | *Taraperla ancilis* | 13.3 (11-15.5) | 11 (9.5-12.5) | 863 (300-1380) | Fully-winged |
|  | *Taraperla howesi* | 13 (12-14) | 12.3 (12.25) | 861 (160-1620) | Fully-winged |
|  | *Taraperla pseudocyrene* | 16.3 (14.5-18) | 10 (10) | 1126 (940-1220) | Fully-winged |
|  | *Taraperla johnsi* | 14 (14) | 12.5 (10-15) | 887 (800-1000) | Wing-reduced |
|  | *Vesicaperla dugdalei* | 15 (13-17) | 12 (12) | 949 (640-1258) | Wing-reduced |
|  | *Vesicaperla eylesi* | unknown | unknown | 1300 (1300-1300) | Wing-reduced |
|  | *Vesicaperla kuscheli* | 20 (20) | 18 (18) | 1250 (1250-1250) | Wing-reduced |
|  | *Vesicaperla substirpes* | 12 (11-13) | 13.2 (13.2) | 1108 (1040-1220) | Micropterous |
|  | *Vesicaperla townsendi* | 18 (18) | unknown | 700 (200-1200) | Wing-reduced |
|  | *Vesicaperla celmisia* | 13 (13) | 12.3 (10.5-14) | 1478 (1250-1600) | Wing-reduced |
|  | *Vesicaperla trilinea* | 18 (18) | 14.5 (14.5) | 1550 (1550-1550) | Wing-reduced |
|  | *Zelandobius alatus* | 6.8 (6-7.5) | 6.8 (5.5-8) | 1366 (980-1680) | Dimorphic |
|  | *Zelandobius albofasciatus* | 10.5 (9-12) | 8.5 (8-9) | 1043 (667-1300) | Fully-winged |
|  | *Zelandobius auratus* | 10 (10) | 9 (9) | 467 (300-600) | Fully-winged |
|  | *Zelandobius brevicauda* | 8 (8) | 7 (7) | 825 (400-1035) | Wing-reduced |
|  | *Zelandobius childi* | 11 (11) | 7.8 (6-9.5) | 1229 (500-1700) | Fully-winged |
|  | *Zelandobius confusus* | 8.3 (6.5-10) | 8 (6.5-9.5) | 520 (20-1600) | Fully-winged |
|  | *Zelandobius cordatus* | 8 (8) | 7 (7) | 868 (400-1280) | Fully-winged |
|  | *Zelandobius crawfordi* | 7.5 (7.5) | 7.3 (7-7.5) | 599 (1125-360) | Fully-winged |
|  | *Zelandobius dugdalei* | 11 (11) | 7 (7) | 1304 (1000-1800) | Fully-winged |
|  | *Zelandobius edensis* | 9 (9) | 8 (7.7-8.2) | 950 (950-950) | Dimorphic |
|  | *Zelandobius edwardsi* | 9.5 (9.5) | 8 (6-10) | 1568 (1050-1800) | Dimorphic |
|  | *Zelandobius foxi* | 11 (9-13) | 7.8 (7.5-9) | 920 (50-1700) | Dimorphic |
|  | *Zelandobius furcillatus* | 6.8 (6.5-7) | 6.3 (6-6.5) | 646 (20-1700) | Fully-winged |
|  | *Zelandobius gibbsi* | 9 (8-10) | 6.5 (6.5) | 1042 (500-1450) | Fully-winged |
|  | *Zelandobius illiesi* | 7.5 (6-9) | 6 (6) | 633 (20-1400) | Fully-winged |
|  | *Zelandobius inversus* | 10 (10) | 9 (9) | 1675 (1675) | Fully-winged |
|  | *Zelandobius jacksoni* | 10 (10) | unknown | 1100 (880-1319) | Fully-winged |
|  | *Zelandobius kuscheli* | 9 (9) | 9 (8) | 783 (150-1240) | Fully-winged |
|  | *Zelandobius macburneyi* | 12.5 (10.5-14.5) | 8 (6-10) | 1265 (70-1830) | Dimorphic |
|  | *Zelandobius mariae* | unknown | 9 (9) | 1610 (1050-1800) | Fully-winged |
|  | *Zelandobius montanus* | 13.5 (13.5) | 10 (10) | 1434 (1000-1950) | Fully-winged |
|  | *Zelandobius ngaire* | 10.5 (10.5) | 10 (10) | 1269 (1158-1380) | Fully-winged |
|  | *Zelandobius patricki* | 9.75 (7-12.5) | 9 (8-10) | 1112 (80-1700) | Fully-winged |
|  | *Zelandobius peglegensis* | 8 (8) | 7 (7) | 936 (800-1320) | Fully-winged |
|  | *Zelandobius pilosus* | 10 (9.3-10.6) | 7.9 (7-8.8) | 752 (240-970) | Fully-winged |
|  | *Zelandobius takahe* | 7 (6.5-7.5) | 7 (7) | 518 (140-920) | Fully-winged |
|  | *Zelandobius truncus* | 7 (7) | 6.5 (6-7) | 604 (200-1450) | Fully-winged |
|  | *Zelandobius unicolor* | 8 (7-9) | 6.8 (6-7.5) | 1082 (500-1725) | Fully-winged |
|  | *Zelandobius uniramus* | 9 (7-11) | 7 (6-8) | 559 (40-1125) | Fully-winged |
|  | *Zelandobius wardi* | 11.5 (11.5) | 9.5 (9.5) | 438 (60-500) | Fully-winged |
|  | *Zelandoperla agnetis* | 12 (10-14) | 10 (9-11) | 439 (30-1220) | Fully-winged |
|  | *Zelandoperla decorata* | 11 (9-13) | 9.3 (8.5-10) | 529 (5-1700) | Fully-winged |
|  | *Zelandoperla denticulata* | 14 (13-15) | 9.5 (9-10) | 349 (10-1020) | Fully-winged |
|  | *Zelandoperla fenestrata* | 18 (14-22) | 12 (10-14) | 894 (30-1900) | Dimorphic |
|  | *Zelandoperla tillyardi* | 17.3 (13.5-21) | 16 (12-20) | 941 (5-1800) | Dimorphic |
| Notonemouridae | *Cristaperla eylesi* | 6.8 (6.5-7 ) | 6 (6) | 755 (10-900) | Fully-winged |
|  | *Cristaperla fimbria* | 6.5 (5.5-7.5) | 5 (4-6) | 757 (100-1600) | Fully-winged |
|  | *Cristaperla waharoa* | 7.5 (7.5) | 6 (6) | 741 (40-1350) | Fully-winged |
|  | *Halticoperla gibbsi* | 6.3 (5.5-7) | 5.5 (5-6) | 1045 (100-1350) | Fully-winged |
|  | *Halticoperla tara* | 6.3 (5.5-7) | 4.8 (4.5-5) | 798 (60-1550) | Fully-winged |
|  | *Halticoperla viridans* | 6.3 (6-6.5) | 4.3 (4-4.5) | 550 (200-1350) | Fully-winged |
|  | *Notonemoura alisteri* | 5.5 (5-6) | 4.5 (4-5) | 554 (75-950) | Fully-winged |
|  | *Notonemoura hendersoni* | 5 (5) | 5.5 (5-6) | 850 (750-950) | Fully-winged |
|  | *Notonemoura latipennis* | 6 (6) | 5.5 (5.5) | 1179 (600-1700) | Dimorphic |
|  | *Notonemoura spinosa* | 6 (6) | 5 (5) | 140 (140-140) | Fully-winged |
|  | *Notonemoura winstanleyi* | 5.5 (5.5) | 5.5 (5.5) | 999 (150-1420) | Fully-winged |
|  | *Omanuperla bruningi* | 6 (6) | 5 (5) | 925 (20-1305) | Fully-winged |
|  | *Omanuperla hollowayae* | 6 (4.5-7.5) | 5 (4-6) | 1110 (940-1250) | Fully-winged |
|  | *Otehiwi sagittarius* | 8.5 (8.5) | 6.5 (6.5) | 880 (880-880) | Fully-winged |
|  | *Spaniocerca acuta* | 7.3 (7-7.5) | 6 (6) | 529 (220-1000) | Fully-winged |
|  | *Spaniocerca bicornuta* | 6.8 (6.5-7) | 5.8 (5.5-6) | 479 (70-1100) | Fully-winged |
|  | *Spaniocerca hamishi* | 7.5 (7-8) | 6.25 (5.5-7) | 300 (120-470) | Fully-winged |
|  | *Spaniocerca longicauda* | 7 (7) | 6 (6) | 769 (75-1600) | Fully-winged |
|  | *Spaniocerca minor* | 7 (6-8) | 7 (7) | 869 (80-1300) | Fully-winged |
|  | *Spaniocerca zelandica* | 9 (8-10) | 7.3 (7-7.5) | 642 (10-1450) | Fully-winged |
|  | *Spaniocerca zwicki* | 7 (7) | 6 (6) | 699 (23-1775) | Fully-winged |
|  | *Spaniocercoides cowleyi* | 5.5 (5.5) | 5.5 (5.5) | 530 (3-1280) | Fully-winged |
|  | *Spaniocercoides foxi* | 5.3 (4.5-6) | 4 (4) | 1183 (980-1370) | Fully-winged |
|  | *Spaniocercoides howesi* | 7 (7) | 5.5 (5.5) | 1226 (400-1800) | Fully-winged |
|  | *Spaniocercoides hudsoni* | 5.3 (5-5.5) | 4.8 (4.5-5) | 283 (40-750) | Fully-winged |
|  | *Spaniocercoides jacksoni* | 4 (4) | unknown | 100 (100-100) | Fully-winged |
|  | *Spaniocercoides philpotti* | 6 (5-7) | 5 (4.5-5.5) | 840 (170-1200) | Fully-winged |
|  | *Spaniocercoides townsendi* | 6 (6) | 5 (5) | 1042 (710-1500) | Fully-winged |
|  | *Spaniocercoides watti* | 6 (6) | 6 (6) | 100 (100-100) | Fully-winged |

**Figure S1.** Mean female body length versus mean male body length for all New Zealand Plecoptera (*r*^2^ = 0.930, *P* < 0.001).


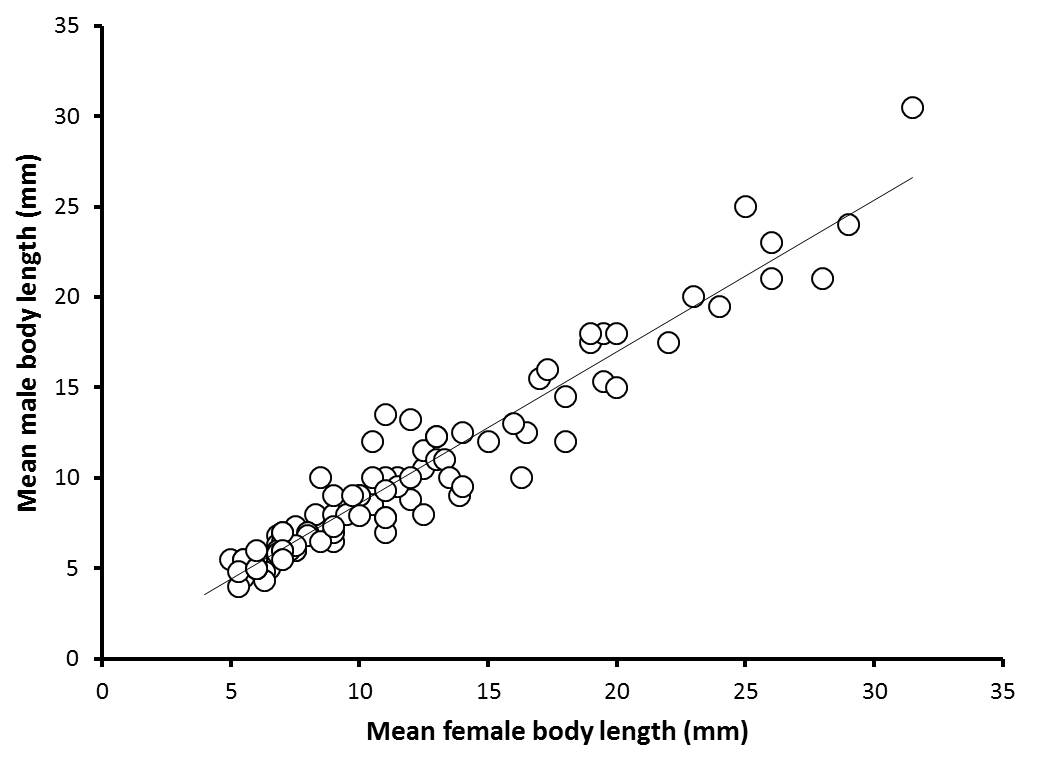


**Appendix S1** Literature sources for body length data of New Zealand Plecoptera.

Gray, D. (2009) A new species of *Zelandobius* (Plecoptera: Gripopterygidae: Antarctoperlinae) from the upper Rangitata River, Canterbury, New Zealand. *New Zealand Journal of Marine and Freshwater Research*, **43**, 605-611.

Illies, J. 1963. The Plecoptera of the Auckland and Campbell Islands. *Records of the Dominion Museum* **4**, 255–265.

Illies, J. 1974. Arthropoda of the subantarctic islands of New Zealand 5. Plecoptera. *New Zealand Journal of Zoology* **1**, 285–294.

Kimmins, D. (1938) LXII.—Notes on the Plecoptera of New Zealand, with descriptions of new species. *Journal of Natural History*, **2**, 561-580.

McCulloch, G.A., Wallis, G.P., Waters, J.M. (2017) Does wing size shape insect biogeography? Evidence from a diverse regional stonefly assemblage. *Global Ecology and Biogeography*, **26**, 93-101.

McLellan, I.D. (1966) Genitalia and nymphs of some New Zealand Gripopterygidae (Plecoptera). *Transactions of the Royal Society of New Zealand - Zoology*, **8**, 5-22.

McLellan, I.D. (1977) New alpine and southern Plecoptera from New Zealand, and a new classification of the Gripopterygidae. *New Zealand Journal of Zoology*, **4**, 119-147.

McLellan, I.D. (1983) A wingless alpine stonefly from New Zealand, and further information on genus *Holcoperla* (Plecoptera: Gripopterygidae). *New Zealand Journal of Zoology*, **10**, 263-266.

McLellan, I.D. (1991) *Notonemouridae (Insecta: Plecoptera)*. Manaaki Whenua Press, Lincoln, New Zealand.

McLellan, I.D. (1993) *Antarctoperline (Insecta: Plecoptera)*. Manaaki Whenua Press, Lincoln, New Zealand.

McLellan, I.D. (1996) A revision of *Stenoperla* (Plecoptera: Eustheniidae) and removal of Australian species to *Cosmioperla* new genus. *New Zealand Journal of Zoology*, **23**, 165-182.

McLellan, I.D. (1997) *Austroperla cyrene* Newman (Plecoptera: Austroperlidae). *Journal of the Royal Society of New Zealand*, **27**, 271-278.

McLellan, I.D. (1998) A revision of *Acroperla* (Plecoptera: Zelandoperlinae) and removal of species to *Taraperla* new genus. *New Zealand Journal of Zoology*, **25**, 185-203.

McLellan, I.D. (1999) A revison of *Zelandoperla* Tillyardi (Plecoptera: Gripopterygidae: Zelandoperlinae). *New Zealand Journal of Zoology*, **26**, 199-219.

McLellan, I.D. (2000) Additions to New Zealand notonemourid stoneflies (Insecta: Plecoptera). *New Zealand Journal of Zoology*, **27**, 21-27.

McLellan, I.D. (2003) Six new species and a new genus of stoneflies (Plecoptera) from New Zealand. *New Zealand Journal of Zoology*, **30**, 101-113.

McClellan, I.D. (2008) Additions to *Zelandobius* (Plecoptera: Gripopterygidae: Antarctoperlinae) from New Zealand. *Illiesia*, **4**, 11-18.

Wisely, B. (1953) Two wingless alpine stoneflies (Order Plecoptera) from southern New Zealand. *Records of the Canterbury Museum*, **6**, 219-231.
